# Multilayer Flow Modulator Stent for Aortic Pathology: A Meta-Analysis and Additional Data from a Single-Centre Retrospective Cohort

**DOI:** 10.31083/j.rcm2503090

**Published:** 2024-03-06

**Authors:** Denise M.D. Özdemir-van Brunschot, Romina Zerellari, Maria Tevs, Sergei Wassiljew, David Holzhey

**Affiliations:** ^1^Department of Vascular Surgery and Endovascular Therapy, Augusta Hospital and Catholic Hospital Group Düsseldorf, 40472 Düsseldorf, Germany; ^2^Faculty of Health, Witten/Herdecke University, 58544 Witten, Germany

**Keywords:** thoracic aneurysm, thoracoabdominal aneurysm, multilayer stent, type B aortic dissection, flow modulator

## Abstract

**Background:**

Thoracoabdominal aneurysms and aortic dissections are a challenge for vascular surgeons. Open surgery, fenestrated or branched endograft, and the chimney technique are not possible in some patients, because of comorbidities or anatomical restrictions. However, the multilayer flow modulator (MFM) can be implanted in some of these patients. In this systematic review, we will describe the experience with the multilayer stent. To augment the limited number of studies available, we will include a cohort of patients from our hospital.

**Methods:**

We retrieved data on all consecutive patients treated using the MFM between May 2013 and August 2020. This included patients with type B dissections and thoracoabdominal or thoracic aneurysms who were unfit for open surgery. The systematic review was performed according to the Preferred Reporting Items for Systematic Reviews and Meta-Analyses (PRISMA) guidelines. We included all the studies that used the MFM in the aortic segment. Single-arm meta-analyses were performed using OpenMeta (Brown University, Providence, RI, USA).

**Results:**

A total of 37 patients were treated in our hospital during the study period. The technical success was 97.3% and the 30-day mortality was 5.4%. In 40.5% of the included patients, the instructions for use were not followed. Off-label implantation was associated with a higher aneurysm-related mortality. A total of 12 studies were included in the meta-analysis and the technical success was 97.8%. In 68.5%, the aneurysm sack or false lumen remained perfused, 97% of all the covered side branches remained patent. After a follow-up period of 1 year, five patients in the meta-analysis presented with a ruptured aneurysm.

**Conclusions:**

The overall quality of evidence is poor because long-term results are lacking, patients are frequently lost during follow-up and all the studies were non-comparative. Our retrospective study suggests a relatively low incidence of perioperative complications, although there was a high incidence of persistent perfusion in the aneurysm sac (102 of 149 patients). The risk of rupture at the 1-year follow-up was 2.1%.

## 1. Introduction

Thoracoabdominal aneurysms and aortic dissections remain challenging for vascular surgeons. Open surgery is not always a viable option due to the complexity of the procedures and the presence of multiple comorbidities in many patients. Fenestrated or branched endografts can be implanted in most patients, however these endografts take time to be manufactured and the procedure has a long learning curve [1]. An alternative is the multilayer flow modulator (MFM, Cardiatis, Isnes, Belgium), which is a self-expanding, non-covered stent made of cobalt alloy. The stent functions by modulating blood flow within the aortic lumen, thereby promoting laminar blood flow. Aneurysms rupture because of increasing stress being applied to the aortic wall at a vulnerable point. Theoretically, redirecting the flow into the laminar flow patterns, promotes flow into side branches and native aorta, thereby reducing the peak wall stress on the aneurysm wall [2]. The MFM does not need to be customized to the specific aortic anatomy of each patient. However, despite the reduction in pressure in the aneurysm, the risk of rupturing still exists.

The use of MFM is a source of debate and controversy after reports of rupture of the aneurysm were presented following device implantation [3, 4, 5, 6, 7]. This skepticism was further fueled by studies reporting continued growth by the treated aortic segments [8, 9]. In this systematic review, we will describe the experience using a multilayer stent. To augment the limited number of studies available, we will include a cohort of patients from our hospital.

## 2. Material and Methods

### 2.1 Additional Hospital Data

To supplement the data for the pooled analysis, we retrieved our data on all consecutive patients treated using the MFM between May 2013 and August 2020. Because five of our patients were also included in the study by Ibrahim *et al*. [8], we excluded these patients from this data set. The study was approved by the local ethics committee (University Witten/Herdecke, S-143/2022), patient´s informed consent was waived.

In our hospital, MFM was used in patients with a type B aortic dissection and thoracoabdominal or thoracic aneurysms who were unfit for open surgery.

A computed tomography (CT) angiography was performed preoperatively. According to the instructions for use, a minimal overlap of at least 6 cm for straight aortas and 8 cm for angulated aortic segments should be used (**Supplementary Table 1**). In addition, there has to be a proximal and distal landing zone of normal arterial wall of at least 2 cm; stenotic side branches have to be treated with stent implantation prior to implantation of the MFM, and stents have to be oversized according to the sizing table in the instructions for use. The instructions for use were not followed in some patients in our cohort. In these cases, there was no other viable alternative due to anatomic restrictions or their comorbidities. In a review by Sultan *et al. * [10], it was also suggested that the MFM was not recommended for an aneurysm diameter of >6.5 cm. In aneurysms with this large diameter, adventitial elastolysis develops, the structural integrity of the aortic wall is lost, and the MFM cannot remodel the aneurysm [10].

Femoral access was obtained using a groin incision or percutaneously. After angiography and under systematic heparinization, a stiff guidewire (Lunderquist, Cook Medical, Bloomington, IN, USA) was positioned in the ascending aorta. Then, the MFM stent was placed through a 20 F introducer sheath. When multiple stents were planned, the stent with the smallest diameter was placed first, followed by the stent with the larger diameter. A remodeling balloon (Reliant; Medtronic, Minneapolis, MN, USA) was only used in patients with an aneurysm or penetrating aortic ulcer. Within 30 days of implantation, a CT angiography (1 mm axial slices) was performed.

Follow-up visits were scheduled after 6 months, and then, yearly thereafter. All patients received postoperative clopidogrel 75 mg daily for at least 12 weeks.

Technical success was defined according to the reporting standards for endovascular aortic repair (EVAR) as the successful introduction and deployment of the device without surgical conversion, mortality, type I or III endoleak, or limb obstruction within the first 24 hours [11]. Clinical success was defined as successful deployment of the endovascular device without aneurysm-related mortality, type I or III endoleak, graft infection, aneurysm expansion (≥5 mm during follow-up), aneurysm rupture or conversion to open repair [11]. MFM has an open cell design and type IA/B and III endoleaks have been described as failure mode I and endoleak type III as failure mode II [10]. Therefore, endoleak type I or III were disregarded in the definition of technical and clinical success.

Acute renal failure was defined as an increase in serum creatinine ≥0.3 mg/dL within 48 hours or an increase in baseline serum creatinine ≥1.5 times or urine volume <0.5 mg/kg/h for 6 hours [12]. We also assessed patient demographics (age, sex, comorbidities, and risk factors), aortic pathology (dissection, penetrating aortic ulcer, aneurysm, and classification), maximal diameter of the aneurysm, previous aortic procedures, number of covered aortic branches, adjuvant procedures, non-adherence to the instructions for use, 30-day outcomes and complications, aneurysm-related outcomes (maximal aneurysm diameter, occluded covered aortic branches, reinterventions because of aneurysm progression, and aneurysm-related death), and all-cause mortality.

Statistical analysis of this retrospective cohort was performed using SPSS (version 27; IBM Corporation, Armonk, NY, USA). Continuous variables were reported as mean and standard deviation and categorical data as absolute numbers and percentages. Statistical significance was defined as *p* < 0.05. Non-normally distributed continuous data were compared using the Mann–Whitney U test; Students’ test was used for normally distributed continuous data. Categorical data were compared using the Pearson χ^2^ test (and Fisher’s exact test when n <5). Kaplan–Meier analysis was used for all-cause and aneurysm-related mortality.

### 2.2 Meta-Analysis

The systematic review was performed according to the Preferred Reporting Items for Systematic Reviews and Meta-Analyses (PRISMA) guidelines [13]. The systematic review was registered at PROPERO (CDR42023454147). The MEDLINE database was systematically searched from January 1st, 2010, to June 1st, 2022 (search terms included [“multilayer” OR “Cardiatis”] AND [“aneurysm” OR aorta*]) and updated on January 1st, 2023. Two authors (DÖ and RZ) confirmed the eligibility of the studies independently. Randomized controlled trials (RCTs), cohort studies, and single-arm studies (including registry studies) were accepted. We only accepted studies in which the MFM (Cardiatis, Isnes, Belgium) was used. Patients with an aneurysm, dissection, penetrating aortic ulcer, false aneurysm, or intramural hematoma of the aorta were included. No limits were applied regarding publication language or status. The references of each identified trial were used to identify any further relevant studies. When multiple studies describing the same population were published, the most complete report was used. Studies that were only available as abstracts were excluded since a quality assessment could not be performed. Studies with <5 patients were also excluded.

The following characteristics were extracted: author and year of publication, country, total number of patients, total number of stents, indication, location of the aortic pathology, number of over-stented side branches, and mean follow-up.

Outcome measures included: technical success, mesenteric ischemia at 30 days, neurological complications at 30 days, patent side branches, thrombosis of the aneurysms or false lumen of the dissection, reintervention at 1 year and maximal follow-up, rupture at 1 year and maximal follow-up, and all-cause mortality at 30 days, 1 year, and maximal follow-up.

Quality assessment of the single-arm studies was performed using the methodological index for non-randomized studies (MINORS) [14]. The MINORS quality assessment contains 12 items, of which the first 8 are specifically for non-comparative studies. MINORS has a high test–retest reliability and good internal consistency [14]. In the assessment of follow-up length, two points were assigned when the mean follow-up length was longer than 12 months and one point when the mean follow-up length was longer than 6 months. The quality assessment was performed by RZ and DÖ.

Since no comparative studies were available, single-arm meta-analyses were performed using OpenMeta (Brown University, Providence, RI, USA). To assess heterogeneity, the $I^{2}$ statistic was used ($I^{2}$ > 75% was used as a threshold indicating significant heterogeneity). All analyses were performed on an intention-to-treat basis. When analyzing complications or reinterventions, the number of complications/reinterventions was used and not the number of patients.

## 3. Results

### 3.1 Additional Hospital Data

A total of 37 consecutive patients were treated using MFM, the demographics of whom are depicted in Table 1. Most of the patients were male (54%). All patients were American Society of Anesthesiologists (ASA) III or IV. A prior aortic procedure was performed in 10 patients, while MFM (off-label) was used in 2 patients to treat a type 1A endoleak.

**Table 1.** **Demographics**.

Mean age (years)		74.8 (SD 7.8)
Male:Female		20:17
Classification		
	Dissection, type B	8 (21.6%)
	Penetrating aortic ulcer	6 (16.2%)
	Aneurysm, descending aorta	2 (5.4%)
	Aneurysm, type I TAAA	4 (10.8%)
	Aneurysm, type II TAAA	1 (2.7%)
	Aneurysm, type III TAAA	4 (10.8%)
	Aneurysm, type IV TAAA	5 (13.5%)
	Aneurysm, type V TAAA	1 (2.7%)
	Juxta/pararenal aneurysm	4 (10.8%)
	Type 1A endoleak	2 (5.4%)
Mean maximal aneurysm diameter		53.0
Previous aortic procedure		
	EVAR	4 (10.8%)
	Thoracic stent	5 (13.5%)
	Ascending aortic replacement	1 (2.7%)
Comorbidities/risk factors		
	Hypertension	37 (100%)
	Diabetes mellitus	6 (16.2%)
	Non-smoking/smoking/ex-smoker	16 (43.2%)/11 (29.7%)/10 (27.0%)
	Hyperlipidemia	19 (51.4%)
	Preoperative dialysis	0 (0%)

SD, standard deviation; EVAR, endovascular aortic repair; TAAA, thoracoabdominal aortic aneurysm.

#### 3.1.1 Perioperative Outcomes

Technical success was achieved in 36 of 37 patients (97.3%) (Table 2). The cause for technical failure was limb occlusion during the first 24-hour postoperative period. This patient was successfully treated with thrombectomy. The MFM covered a total of 125 arteries, whereby 121 arteries remained patent during the study period. In one patient, a nephrectomy was necessary because of malignant hypertension after the occlusion of the renal artery. A second patient was treated with an iliac–renal bypass after acute occlusion of the renal artery. Another patient presented to the outpatient clinic with renal artery occlusion 14 months after MFM and was treated conservatively. A fourth patient died because of mesenteric ischemia after occlusion of the superior mesenteric artery (the celiac axis was occluded preoperatively).

**Table 2.** **Procedure details**.

		Number of events (%)
Technical success		36 (97.3%)
Number of covered aortic branches		125
	Brachiocephalic artery	6 (4.8%)
	Left common carotid artery	6 (4.8%)
	Left subclavian artery	11 (8.8%)
	Celiac axis	27 (21.6%)
	Superior mesenteric artery	24 (19.2%)
	Left renal artery	24 (19.2%)
	Right renal artery	27 (21.6%)
Other procedures		
	Coiling of the aneurysm sack	1 (2.7%)
	Stent implantation aortic branch	5 (13.5%)
	Covered stent prosthesis	6 (16.2%)
	Other	1 (2.7%)
Non-adherence to the instructions for use		15 ${\mathrm{(40.5\%)}}^{1}$
	Overlap	3 ${\mathrm{(8.1\%)}}^{2}$
	Non-aneurysmal landing zone	2 ${\mathrm{(5.4\%)}}^{2}$
	Stenotic artery not preoperatively stented	1 ${\mathrm{(2.7\%)}}^{2}$
	Previously implanted aortic stent or aortic graft	10 ${\mathrm{(27.0\%)}}^{2}$
	Improperly sizing	2 ${\mathrm{(5.4\%)}}^{2}$

^1^Number of patients in whom the instructions for use were not adhered to. ^2^Number of violations (in some patients more than one violation was present).

The 30-day complication rate was 13.5% (5/37) (Table 3). This included the previously reported patient who died because of mesenteric ischemia, the patient with limb occlusion, and the patient who was treated with a nephrectomy due to occlusion of the renal artery. One patient suffered from a postoperative stroke, he initially survived but died 10 months later from a ruptured aneurysm. Another patient died because of a retrograde type A dissection, which occurred after deployment of the MFM. The 30-day mortality was 5.4% (2/37).

**Table 3.** **Outcome**.

Outcome at 30-days		
	Aneurysm-related death	1 (2.7%)
	Neurological complications	1 (2.7%)
	Embolization	1 (2.7%)
	Number of patent-covered aortic branches	123/125 (98.4%)
	Leg ischemia	1 (2.7%)
	Stenosis aortic branch	1 (2.7%)
	Acute renal failure	0 (0.0%)
Clinical success at 30 days		36 (97.3%)
Postoperative outcomes		
	Number of patent-covered aortic branches at 1 year	122/125 (97.6%)
	Number of patent-covered aortic branches	121/125 (96.8%)
	Mean maximal aneurysm diameter (mm)	58.2
	Perfusion of aneurysm sack/false lumen	31 (83.8%)
	Stenosis of covered aortic branch	1 (2.7%)
	Leg ischemia	2 (5.4%)
	Reintervention due to aneurysm progression	8 (21.6%)
	Aneurysm-related death	7 (18.9%)

#### 3.1.2 Postoperative Outcomes

**Fig. 1.**
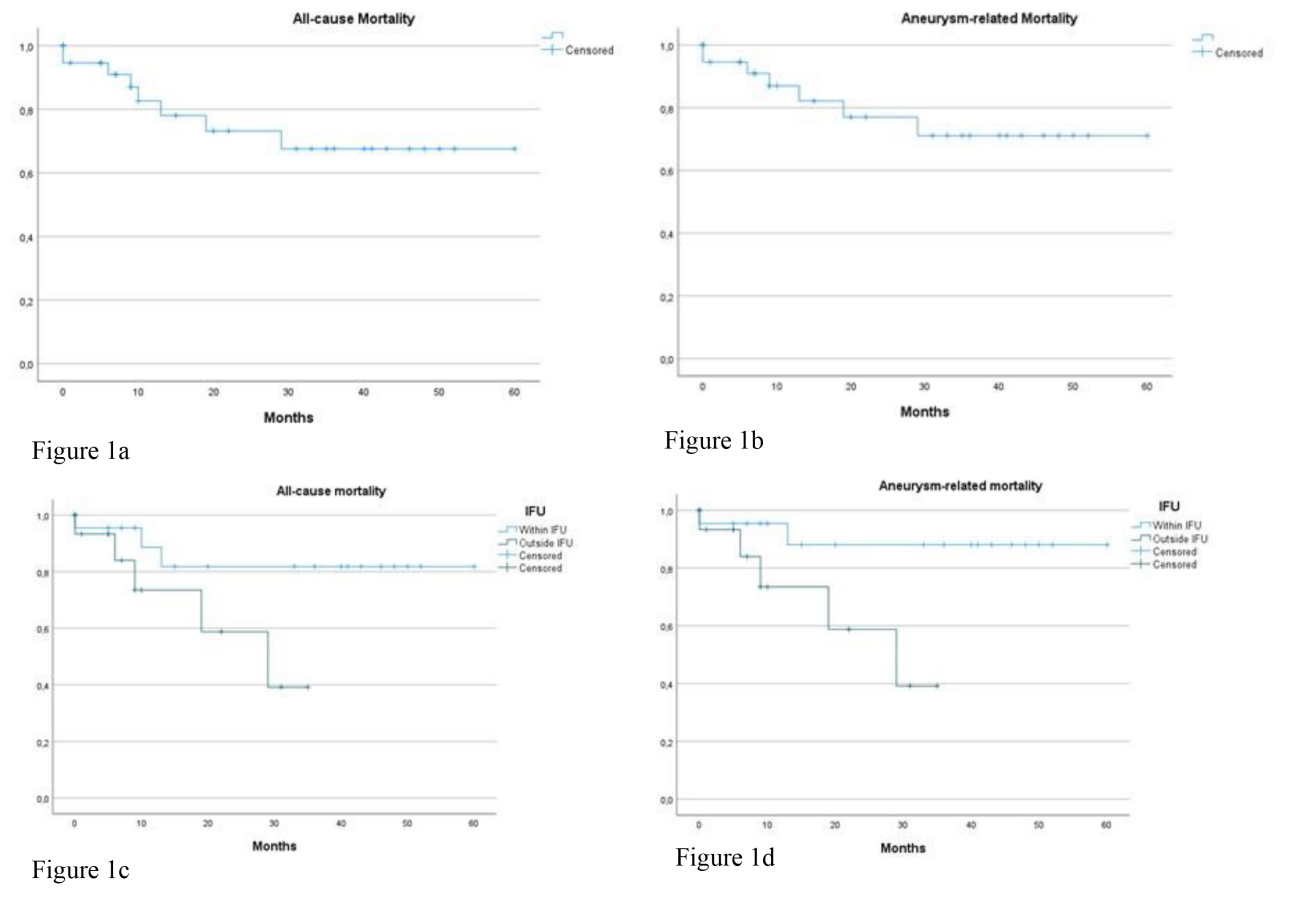
**All-cause and aneurysm-related mortality**. (a) All-cause mortality. (b) Aneurysm-related mortality. (c) All-cause mortality: within and outside IFU. (d) Aneurysm-related mortality: within and outside IFU. IFU, instructions for use.

The mean follow-up was 19.4 months (SD 18.4). In 83.8% of patients with an aneurysm, the aneurysm sack or the false lumen (in patients with a dissection) remained perfused (Table 2). The maximal diameter of the aneurysm in patients increased from 53.0 mm to 58.2 mm. All-cause mortality and aneurysm-related mortality are displayed in Fig. 1. There was a trend towards higher overall survival rates in treated patients when the instructions for use were followed (*p *= 0.08); aneurysm-related survival was significantly improved in patients treated within the instructions for use (*p *= 0.03).

A reintervention was necessary in eight (21.6%) patients due to the progression of the aneurysm sack, table 3. In seven patients, an additional MFM or thoracic endovascular aortic repair ((T)EVAR) was implanted because of mode I or III failure, in one patient a debranching procedure was performed. Other reinterventions included stenting of aortic side branches (*n *= 1), nephrectomy (*n* = 1), iliac-renal bypass (*n* = 2), and thrombectomy (*n* = 1).

### 3.2 Meta-Analyses

**Fig. 2.**
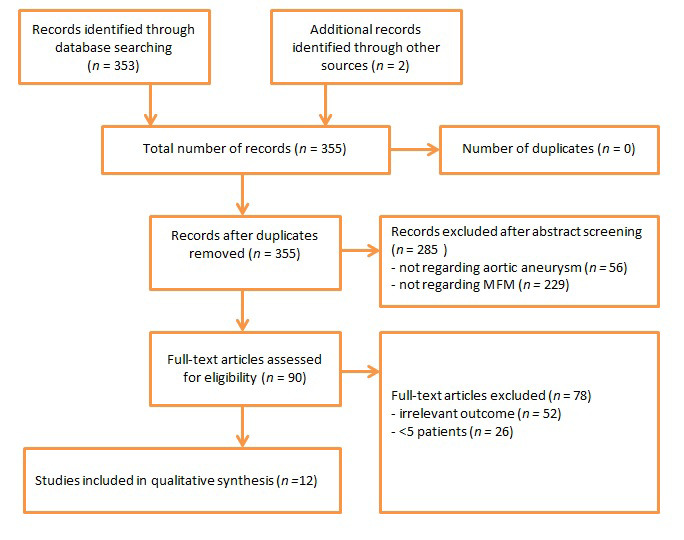
**Study flow chart**. MFM, multilayer flow modulator.

A total of 353 potentially eligible studies were identified (Fig. 2). After screening the titles and abstracts for inclusion, a total of 12 studies met the inclusion criteria. The characteristics of the included studies are depicted in Table 4 (Ref. [4, 8, 9, 15, 16, 17, 18, 19, 20, 21, 22, 23]). There were five registered studies describing the same patient population [15, 17, 23, 24, 25], the study with the most patients and most complete follow-up data was included in the meta-analysis [23].

**Table 4.** **Studies included in the meta-analyses**.

	Time period	Number of patients	Number of male patients	Country	Indication
Benjelloun *et al*., 2016 [18]	June 2009 to September 2012	18	16	Morocco	Thoracoabdominal aortic aneurysm and abdominal aortic aneurysm
Bouayed *et al*., 2016 [19]	March 2023 to December 2013	38	25	Algeria	Thoracoabdominal aortic aneurysm, descending thoracic aortic aneurysm, juxta/infrarenal aneurysm, and aortic dissecting hematoma
Costache *et al*., 2021 [15]	April 2014 to February 2019	23	20	Romania	Type B aortic dissection
Debing* et al*., 2014 [4]	March 2012 to December 2012	6	4	Belgium	Aortic arch aneurysm, thoracoabdominal aortic aneurysm, and juxtarenal aneurysm
Ibrahim *et al*., 2018 [8]	January 2009 to June 2014	40	29	Germany	Thoracoabdominal aortic aneurysm, descending thoracic aortic aneurysm, juxtarenal aneurysm, pararenal aneurysm, para-anastomotic aneurysm, infrarenal aneurysm, and penetrating atherosclerotic ulcers
Lowe *et al*., 2016 [9]	October 2011 to March 2014	14	10	United Kingdom	Thoracoabdominal aortic aneurysm, juxta/suprarenal aneurysm, and saccular arch aneurysm
Ovali* et al*., 2018 [20]	April 2014 to February 2016	23	19	Turkey	Thoracoabdominal aortic aneurysm and iliac aneurysm
Pane *et al*., 2016 [21]	November 2011 to November 2012	8	6	Italy	Thoracoabdominal aortic aneurysm, juxtarenal aneurysm, and iliac aneurysm
Polydorou * et al*., 2012 [22]	December 2006 to December 2011	22	22	Greece	Descending thoracic aortic aneurysm, thoracoabdominal aortic aneurysm, and abdominal aneurysm
Sultan *et al*., 2014 [23]	n.s.	103	74	12 countries	Thoracoabdominal aortic aneurysm, arch aneurysm, suprarenal aortic aneurysm, and type B dissection
Wang *et al*., 2020 [16]	May 2012 to December 2015	8	7	China	Thoracoabdominal aortic aneurysm
Vaislic *et al*., 2014 [17]	April 2010 to February 2011	23	19	France	Thoracoabdominal aortic aneurysm
Hospital cohort, 2023	May 2013 to August 2020	37	20	Germany	Type B dissection, descending thoracic aortic aneurysm, thoracoabdominal aortic aneurysm, juxta/pararenal aneurysm, and type 1A endoleak

n.s., not stated.

In most studies, a thoracoabdominal aneurysm was the indication used for MFM implantation. However, only patients with an aortic dissection were included in one study [15]. Since no comparative studies could be included, the quality assessment was based on the first eight questions in the MINORS quality assessment tool (**Supplementary Table 2**). In none of the studies, the study size was prospectively calculated, meaning the maximal score of 16 was not reached in the quality assessment. In addition, the outcome assessments were not performed by an unbiased researcher in most studies.

**Fig. 3.**
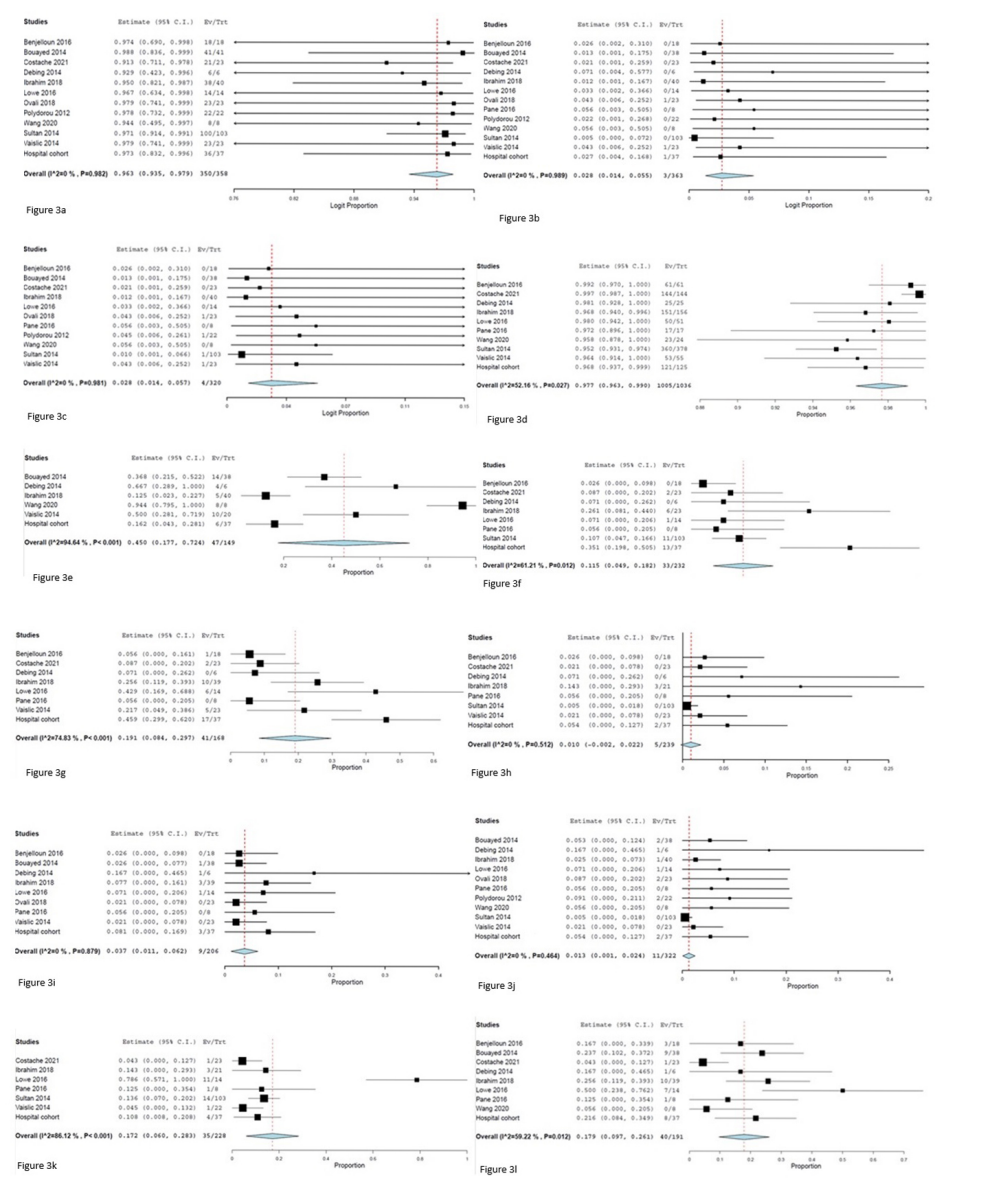
**Meta-analyses**. (a) Technical success. (b) Mesenteric ischemia at 30 days. (c) Neurological complications at 30 days. (d) Patency of side branches at maximal follow-up. (e) Complete thrombosis of the aneurysm sac at maximal follow-up. (f) Reinterventions at 1 year. (g) Reinterventions at maximal follow-up. (h) Rupture at 1 year. (i) Rupture at maximal follow-up. (j) All-cause mortality at 30 days. (k) All-cause mortality at 1 year. (l) All-cause mortality at maximal follow-up. Ev, event; Trt, treated patient.

Outcomes are shown in Fig. 3 and indicate a technical success of 97.8% (350/358). At 30 days, three cases of mesenteric ischemia (0.8%) and four cases of neurological complications (1.2%) were identified. Neurological complications included one case of a transient ischemic cerebrovascular event, two patients who suffered a stroke, and one case of a hemorrhagic cerebrovascular stroke. Most covered aortic side branches remained patent during the follow-up study (1005 of 1036, 97.0%). Additionally, the aneurysm sack or false lumen remained perfused in 68.5% (102 of 149) of the patients during the follow-up study. Reinterventions were necessary for 14.2% of patients (33 of 232) after 1 year and 24.4% (41 of 168) at the maximal follow-up. The incidence of aneurysm rupture was 2.1% (5 of 539) at 1 year and 4.4% (9 of 206) at the maximal follow-up. All-cause mortality was 3.4% (11 of 322) at 30 days, 15.4% at 1 year (35 of 228), and 20.9% (40 of 191) at the maximal follow-up (mean of 14.3 months).

## 4. Discussion

When compared to fenestrated aortic stents, MFM implantation is a less demanding procedure, which does not require catheterization and stenting of side branches, thereby reducing total fluoroscopy time and contrast material volume. This was reflected by the high technical success rate (97.3% in our hospital cohort and 97.8% in the meta-analysis).

However, the use of MFM is controversial due to reports of persistent perfusion of the aneurysm sack or false lumen, and thus, a persistent risk of aneurysm rupture. In this review, the incidence of rupture at 1 year was 2.1%, while it was 4.4% at the maximal follow-up. There were notable differences in the incidence of thrombosis of the aneurysm sack or false lumen in the included studies (Fig. 3e). In some studies, complete thrombosis of the aneurysm sack was achieved in all patients, whereas in other studies, including our own cohort, complete thrombosis was achieved in less than 20% of all patients. A possible reason for this heterogeneity could be differences in off-label use. In our study, the MFM was used off-label in 40.5% of all patients. In the study by Ibrahim *et al*. [8], 12 of 40 patients received the MFM outside the instructions for use. Thrombosis of the aneurysm sack or false lumen was rarely encountered in either study. Unfortunately, the number of patients in which the MFM was used off-label was not provided in all the studies [4, 16, 17]. The deleterious effect of MFM implantation off-label was described in a study by Sultan *et al*. [24]. The study group described an all-cause mortality of 89.5% during a mean follow-up of 10.0 months, where 71.1% was aneurysm-related.

A major limitation of this study is its single-arm design. However, finding a suitable control group was challenging since the MFM is only used in patients when the use of both open and endovascular (*e.g.*, fenestrated or branched devices) techniques were not feasible. Perioperative mortality in open repair of thoracoabdominal aneurysms is approximately 9% [25], although in some studies it is reported to be as high as 20% [26, 27]. In a systematic review, the perioperative mortality in the endovascular and open repair of thoracoabdominal aneurysms was comparable [25]. The pooled perioperative mortality in endovascular repair was 7.4% in this meta-analysis [25]. Another option is hybrid (“debranching”) repair of thoracoabdominal aneurysms. However, this approach is associated with mortality and morbidity rates as high as those for open repairs [28]. In this study, the pooled 30-day mortality was 3.4%, which is lower than for open, and complex endovascular and hybrid repairs.

However, the relatively low 30-day mortality comes at a price since the mortality at 1 year and at the maximal follow-up (mean 14.3 months) in this systematic review was 15.4% and 20.9%, respectively. Studies on open thoracoabdominal aortic aneurysm (TAAA) repair have reported midterm survival rates for this procedure between 83% and 90% [29, 30, 31]. Endovascular TAAA repair is associated with a midterm survival of approximately 88% [32, 33]. Although there are no comparative studies, it appears that the overall mortality of the MFM is worse when compared to open and endovascular TAAA repairs; however, it must be noted that the MFM is only used in patients with no other alternatives.

Another limitation is the short follow-up period that was found in all the included studies. The mean follow-up of the included studies was 14.3 months. This can partly be explained by the high percentage of patients that were lost during the follow-up period. A study by Schanzer *et al*. [34] described a loss during follow-up after EVAR of 22% at 1 year, 38% at 3 years, and 50% at 5 years. A possible explanation could be that most patients have multiple comorbidities and may lose focus on surveillance. In our hospital cohort, 3 (8.1%) patients did not come to the outpatient clinic after discharge. Frequent follow-ups are important because the incidence of major adverse events is higher in patients without frequent follow-ups [35].

Another important limitation was the high incidence of heterogeneity in some of the included meta-analyses. High levels of heterogeneity were detected when describing the incidence of thrombosis of the aneurysm sack, reinterventions, and all-cause mortality. This is most likely due to differences in off-label use.

Violations of the instructions for use can have deleterious consequences for patients treated with the MFM stent. In our cohort study, the overall- and aneurysm-related survival was higher for patients treated within the instructions for use (*p* = 0.08 and *p* = 0.03). Likewise, other studies have reported comparable results. Sultan *et al*. [24] described 38 patients who had been treated outside the instructions for use. Aneurysm-related mortality was 74.8% at 18 months, which is a finding that was confirmed in another systematic review [36]. Aneurysm-related survival at 1 year was 93.3% for patients treated within the instructions for use, whereas aneurysm-related survival at 1 year was 38.0% for patients treated outside the instructions for use.

## 5. Conclusions

The overall quality of existing studies that used MFM was poor. All studies are performed as non-comparative studies since finding a suitable comparison group is difficult, long-term results are lacking, and patients are frequently lost during the follow-up period. In our opinion, MFM should only be considered in patients without other available treatment options and when it is possible to adhere to the instructions for use. In addition, the disadvantages should be discussed with the patient.

## Abbreviations

ASA, American Society of Anesthesiologists; EVAR, endovascular aortic repair; MFM, multilayer flow modulator.

## Supplementary Material

Supplementary material associated with this article can be found, in the online version, at https://doi.org/10.31083/j.rcm2503090.
