# Supplementary material for: Multilayer Flow Modulator Stent for Aortic Pathology: A Meta-Analysis and Additional Data from a Single-Centre Retrospective Cohort
Source: Rev Cardiovasc Med. 2024 Mar 6;25(3):90. doi: 10.31083/j.rcm2503090 (PMC11263838; doi:10.31083/j.rcm2503090)
Supplement: Supplementary file 1 [file 2153-8174-25-3-090-s1.zip › Supplementary material.pdf]

Supplementary Table 1: Instructions for use

**Contraindications**	**Warnings and precautions**
Arteriovenous fistula<br><br>Inadequate distal flow<br><br>Inadequate arterial access (due to tortuosity, calcifications or occlusions)<br><br>History of coagulation problems<br><br>Suspected infection (e.g. mycotic aneurysm)<br><br>Presence/suspicion of connective tissue disorder or Takayasu's arteritis<br><br>Pregnant or breastfeeding woman<br><br>Persons aged 18 and under<br><br>Shaggy aorta<br><br>Patients who cannot tolerate contrast agents<br><br>Ruptured aneurysms<br><br>Aortic root aneurysm<br><br>Previously implanted stent grafts<br><br>History of coagulation problems	When covering aortic branches with stenosis or thrombus, these branches should be preoperatively stented<br><br>The stent with the bigger diameter should be placed after the implantation of the stent with the smaller diameter<br><br>For sizing: adherence to the MFM sizing table<br><br>Stents should have an overlap of minimal 6 cm in straight segment and minimal 8 cm in curved segments<br><br>The proximal and distal landing zones (non-aneurysmatic) should be at least 2 cm

Supplementary Table 2: MINORS Quality assessment

	Clearly stated aim	Inclusion of consecutive patients	Prospective collection of data	Endpoints appropriate to the aim of the study	Unbiased assessment of the study endpoint	FU period appropriate to the aim of the study	Loss to FU less than 5%	Prospective calculation of study size	Total
Benjelloun, 2018 (32)	2	2	2	2	0	1	1	0	11
Bouayed, 2016 (33)	2	0	2	2	0	1	2	0	9
Costache, 2021 (16)	2	2	2	2	0	1	2	0	11
Debing, 2014 (4)	2	0	2	2	2	1	2	0	11
Ibrahim, 2018 (8)	2	0	0	2	0	2	1	0	7
Lowe, 2016 (9)	2	0	0	2	0	2	2	0	8
Ovali, 2018 (34)	2	0	0	2	0	2	2	0	8
Pane, 2016 (35)	2	0	0	2	0	2	2	0	8
Polydorou, 2012 (36)	2	0	0	0	0	2	0	0	4
Sultan, 2014 (15)	2	1	2	2	0	1	1	0	9
Wang, 2020 (17)	2	0	0	2	2	2	2	0	10
Vaislic, 2014 (18)	2	0	2	2	0	1	0	0	7
